# Genetic mapping uncovers *cis*-regulatory landscape of RNA editing

**DOI:** 10.1038/ncomms9194

**Published:** 2015-09-16

**Authors:** Gokul Ramaswami, Patricia Deng, Rui Zhang, Mary Anna Carbone, Trudy F. C. Mackay, Jin Billy Li

**Affiliations:** 1 Department of Genetics, Stanford University, Stanford, California 94305, USA; 2 Department of Biological Sciences, Program in Genetics and W. M. Keck Center for Behavioral Biology, North Carolina State University, Raleigh, North Carolina 27695, USA

## Abstract

Adenosine-to-inosine (A-to-I) RNA editing, catalysed by ADAR enzymes conserved in metazoans, plays an important role in neurological functions. Although the fine-tuning mechanism provided by A-to-I RNA editing is important, the underlying rules governing ADAR substrate recognition are not well understood. We apply a quantitative trait loci (QTL) mapping approach to identify genetic variants associated with variability in RNA editing. With very accurate measurement of RNA editing levels at 789 sites in 131 *Drosophila melanogaster* strains, here we identify 545 editing QTLs (edQTLs) associated with differences in RNA editing. We demonstrate that many edQTLs can act through changes in the local secondary structure for edited dsRNAs. Furthermore, we find that edQTLs located outside of the edited dsRNA duplex are enriched in secondary structure, suggesting that distal dsRNA structure beyond the editing site duplex affects RNA editing efficiency. Our work will facilitate the understanding of the *cis*-regulatory code of RNA editing.

## Abstract (web summary)

Adenosine-to-inosine (A-to-I) RNA editing plays an important role in neurological functions. Here, by a quantitative trait loci (QTL) mapping approach in 131 *Drosophila melanogaster* strains, the authors identify 545 QTLs associated with differences in RNA editing.

## Graphical abstract

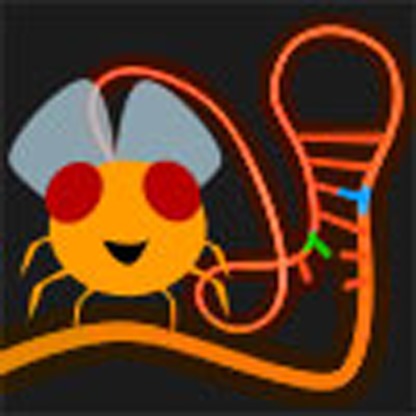

RNA editing is the modification of RNA nucleotides from their genome-encoded sequence. The most common type of RNA editing in metazoans is the deamination of adenosine into inosine catalysed by the adenosine deaminase acting on RNA (ADAR) family of enzymes^1^. Inosine is recognized as guanosine by the cellular machinery and A-to-I editing in coding sequences often leads to amino acid changes in proteins. A-to-I editing is prevalent in the fruit fly *Drosophila melanogaster* (*D. melanogaster*), with over 5,000 RNA editing sites identified^2^,^3^,^4^,^5^. ADAR proteins perform critical neurological functions^6^. In *Drosophila*, knockout of the *ADAR* gene results in severe neurological phenotypes including impaired locomotion, defective flight and male mating difficulties^7^.

A-to-I editing occurs cotranscriptionally in the nucleus when double-stranded RNA (dsRNA) is formed at the pre-mRNA level, which is subsequently bound and edited by ADARs^1^,^8^. Perfect dsRNA duplexes such as those formed by primate *Alu* repeats are promiscuously edited^9^; however in non-repetitive sequences, imperfect dsRNA structures are formed and editing only occurs at specific adenosines^10^. The mechanisms whereby ADAR targets a specific non-repetitive A-to-I RNA editing site are not well understood. Both the primary sequence and secondary structure (that is, *cis*-acting regulatory elements) surrounding the editing site guide the preference and selectivity of ADARs. ADAR has a preferred sequence motif, in particular the 5′ and 3′ nearest neighbouring positions to the editing site^11^,^12^,^13^. Additionally, adenosines edited in a dsRNA are affected by mismatches, bulges and loops, implicating complex structural contributions to editing specificity^12^,^14^. Distal tertiary structures have also been shown to influence RNA editing efficiency in two transcripts^15^,^16^.

Quantitative trait loci (QTL) mapping in natural populations is a strategy that has been used to successfully study the regulatory architecture of many molecular phenotypes such as gene expression (eQTLs)^17^,^18^ and splicing patterns (sQTLs)^19^,^20^. To characterize rules governing ADAR targeting specificity, we measure the variation in RNA editing within a natural population of *D. melanogaster* and identify genetic variants that are associated with changes in editing levels. Using these RNA editing QTL (edQTL), we examine how changes in RNA secondary structure induced by genetic variants affects RNA-editing levels.

## Results

### Quantifying RNA editing in the DGRP

**Figure 1:**
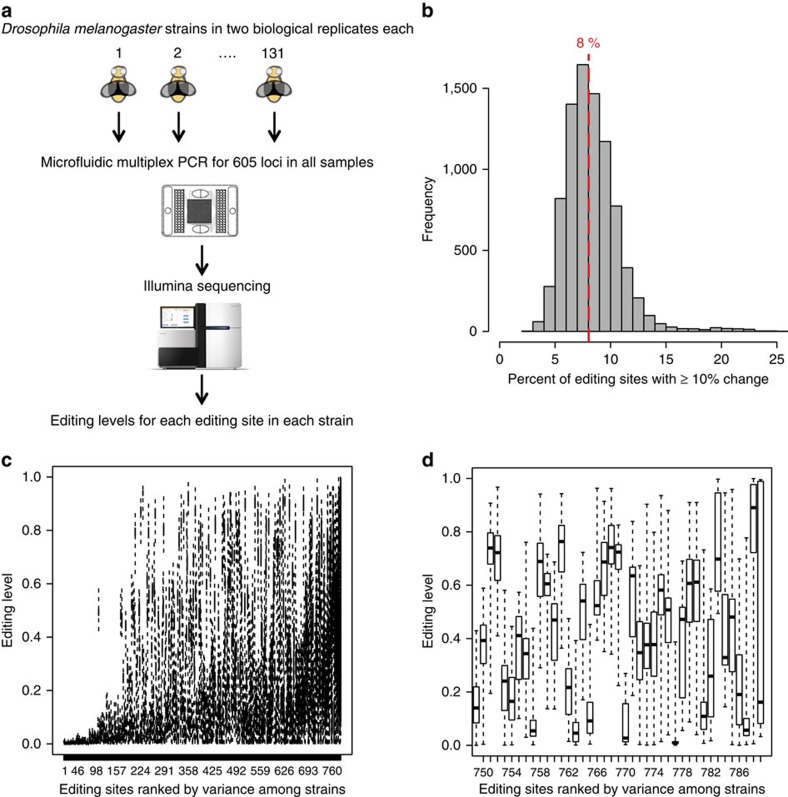
Quantifying RNA editing in the DGRP. (**a**) Overview of experimental scheme. We used mmPCR-seq to quantify RNA editing levels in 789 sites within 605 loci in 131 *D. melanogaster* strains. (**b**) Percentage of editing sites that vary between strains. For each pairwise comparison between strains, the fraction of editing sites that have a 10% or greater editing level difference between the two strains is plotted. The red dashed line corresponds to the median value of 8%. (**c**,**d**) Editing variability in DGRP strains. Editing levels for (**c**) all 789-editing sites and (**d**) the 40 most variable editing sites are plotted. Editing sites are ranked by binomial variance, a metric of variability in the 131 strains.

To study natural variation of RNA editing in *D. melanogaster*, we quantified RNA editing levels in replicate from male whole bodies in 131 strains of the Drosophila Genetic Reference Panel (DGRP) using mmPCR-seq, an efficient method we recently developed^21^ (Fig. 1a). Publically available genotypes were available for all of the DGRP strains^22^. The mmPCR-seq assay utilizes the Fluidigm Access Array microfluidic chip to PCR amplify 605 loci (Supplementary Data 1) for each sample separately, and then barcodes each sample before deep sequencing the amplified products^21^. After mapping the sequencing reads onto the genome, RNA editing levels are calculated as the fraction of reads containing a ‘G' nucleotide at each RNA editing site. We observed a high concordance between replicates, verifying the robustness of mmPCR-seq (Supplementary Fig. 1). After filtering editing sites in areas of low coverage (Methods section), we are left with a data set of 789 editing sites measured in at least 35 strains (Supplementary Fig. 2) to be used for QTL mapping.

A-to-I RNA editing is heavily clustered and we attempted to identify additional RNA editing sites using our mmPCR-seq data, an approach previously demonstrated in human samples^21^. By performing *de novo* identification of RNA editing sites in each sample, we identified 1,202 novel A-to-I RNA editing sites with an estimated false discovery rate of <2% (Supplementary Fig. 3). However, an overwhelming majority, 95% of novel RNA editing sites were edited <5% and we did not include these novel RNA editing sites in our subsequent analyses.

RNA editing levels generally tend to be low at most editing sites, with 51% of all RNA editing levels <10%, 35% between 10 and 50%, and 14%>50%. Using hierarchical clustering, we did not observe large global differences in RNA editing between strains (Supplementary Fig. 4). However, we did observe considerable modest differences with an average of 8% of the editing sites having a 10% or greater editing level difference between pairs of strains (Fig. 1b) and many individual editing sites having considerable variability in editing levels between the 131 strains (Fig. 1c,d).

### Association of RNA editing with genetic variants

**Figure 2:**
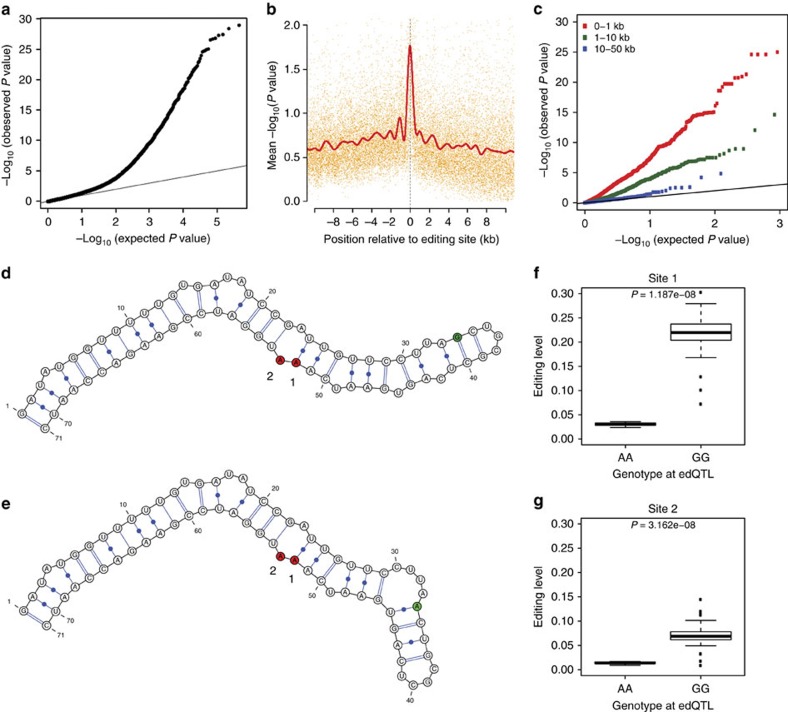
Mapping RNA editing QTLs. (**a**) Quantile–quantile (QQ) plot for association testing *P* values between 789 RNA editing sites and genetic variants in the same gene as each editing site. (**b**) Significance of association tests in relation to the distance between the editing site and variant. The solid red line is a cubic smoothing spline fit to the data. Transcripts were oriented such that negative and positive values represent variants transcriptionally up- and downstream of the editing site, respectively. (**c**) QQ plot for association tests between 545 edQTLs and additional editing sites that fell within 1 kb (red), between 1 kb and 10 kb (green), and between 10 kb and 50 kb (blue) from the original best-associated editing site. (**d**–**g**) Example of an RNA editing QTL in the *CROL* gene. Predicted local RNA secondary structure for the (**d**) *G* and (**e**) *A* alleles. Two editing sites influenced by the edQTL are shaded in red (numbered 1 and 2) and the edQTL is shaded in green. Relationship between editing levels and strain genotypes for the edQTL at the two associated editing sites (linear model), (**f**) chr2L:11796345 (site 1) and (**g**) chr2L:11796346 (site 2).

To identify genetic variants that could explain the inter-strain variability of RNA editing, we ran association tests between editing levels and genotypes for all variants genome-wide at each editing site (Supplementary Fig. 5). We found that almost all variants meeting a genome-wide significance threshold were located close to their associated editing site and acting in cis. To enhance our power to identify *cis*-edQTLs, we reran the association tests but restricted the variant search space to only those within the same gene as each editing site (Fig. 2a). For each editing site, we ran permutations to calculate an empirical *P* value (Methods section) for the top associated variant and found an abundance of very low *P* values (Supplementary Fig. 6a). We identified 422 and 353 primary RNA editing QTLs (edQTLs) at false discovery rates (FDRs) of 10 and 5%, respectively (Supplementary Data 2). To identify additional variants associated with RNA editing, we regressed out the effect of each primary edQTL and reran the association tests and permutations (Supplementary Fig. 6c). We identified 123 and 114 secondary edQTLs at FDRs of 10 and 5%, respectively (Supplementary Data 2). We observed that edQTLs tend to be present for editing sites with greater variance in editing levels between the 131 strains (Supplementary Fig. 7).

We observed that variants within 1 kb of editing sites were more likely to have significant associations (Fig. 2b). Indeed the edQTLs identified were highly enriched within 5 kb of their associated editing site (Supplementary Fig. 6b,d) with 285 (52%) being within 1 kb. We reasoned that due to the propensity of edQTLs to be located close to their associated editing site, they should also influence additional editing sites nearby. This reasoning was strengthened by the observation that editing levels of editing sites within the same gene are more closely correlated than editing levels of editing sites in different genes and furthermore that editing levels of editing sites within the same RNA duplex are most correlated (Supplementary Fig. 8). We tested the association of edQTLs with all other RNA editing sites in the same gene and found strong associations with additional editing sites within 1 kb of the original most strongly associated editing site (Fig. 2c), demonstrating a shared regulatory mechanism.

### Prediction of editing complementary sequences

RNA editing QTLs tend to be close to their associated editing site and their likely mechanism of action is through changes in local RNA structure, demonstrated by an edQTL in the *CROL* gene (Fig. 2d–g). To characterize how edQTLs affect RNA structure, we needed to first predict the local RNA structure around editing sites. Editing occurs within dsRNA structures in which the editing site stem base-pairs with an editing complementary sequence (ECS)^1^. The ECS is thought to be required for editing, but, to date, only a handful of predicted ECSs have been reported^23^,^24^,^25^,^26^. Finding ECSs in the pre-mRNA is difficult because they can be proximal or distal to the editing sites and many lie in intronic regions.

**Figure 3:**
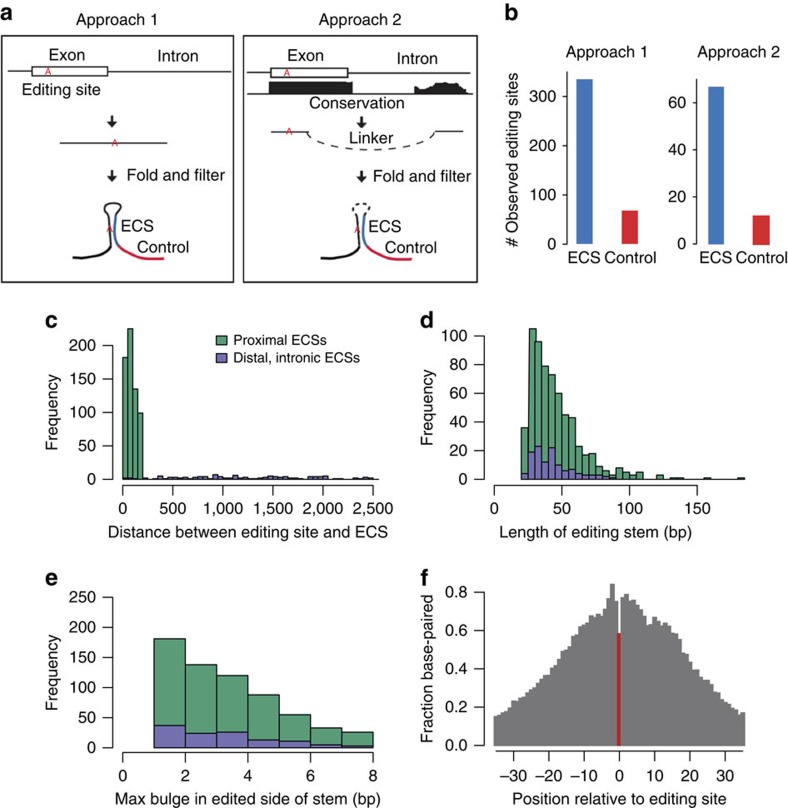
Prediction of ECSs. (**a**) Overview of approaches to predicting proximal ECSs (Approach 1) and distal, intronic ECSs (Approach 2). (**b**) Numbers of editing sites identified in predicted ECSs (blue) compared with the control regions (red) of the same length indicated in (**a**). (**c**) The distribution of the distances between the editing site and the corresponding base in the ECS. (**d**,**e**) The distribution of (**d**) stem lengths and (**e**) max bulge sizes in the editing site side of the stem. The colour legend is the same as in (**c**). (**f**) The fraction of editing substrates that are base-paired at each position relative to the editing site (position 0, indicated in red), based on the predicted editing substrate structure. Negative positions are upstream (5′) of the editing site; positive positions are downstream (3′).

We developed two complementary computational approaches to predict ECSs for editing sites genome-wide (Fig. 3a). To determine the optimal parameters and estimate the accuracy of ECS predictions, we relied on the fact that ECS regions, as one stem of the dsRNA structure, are likely to be edited^8^. We developed an enrichment score metric to calculate the ratio of RNA editing sites in the ECS to those in the flanking regions of the same length. However, the majority of *D. melanogaster* editing sites identified from polyA+ RNA-seq data lie in exonic regions and we would not be able to predict ECS locations in introns using the existing list of RNA editing sites. To overcome this limitation, we applied a highly sensitive RNA editing identification method we recently developed^27^ to the *D. melanogaster* nascent RNA-seq data^8^ and identified a total of 6,566 intronic RNA editing sites (Supplementary Data 3), of which 5,970 (91%) were novel (Supplementary Fig. 9).

In Approach 1, we predicted proximal ECSs by folding the sequence of the region within 200 bps up- and downstream of the editing sites (Methods section) and found ECSs for 641 editing sites (Supplementary Data 4). We defined a sequence as an ECS if there was a dsRNA structure containing the RNA editing site having a stem of at least 20 bp and a max bulge size of 8 bp. These cutoffs were selected because they generate predicted ECSs with the highest enrichment score and a relatively high sensitivity compared with other cutoffs (Supplementary Fig. 10). In Approach 2, we predicted distal, intronic ECSs by folding the region surrounding editing sites with candidate conserved intronic regions (Methods section) and found ECSs for 119 editing sites (Supplementary Data 4), including all seven previously determined intronic ECSs in *Drosophila* (Supplementary Table 1). We observed a fivefold enrichment of editing in the predicted ECS regions as compared with the flanking control regions (Fig. 3b). This suggests high accuracy of our ECS predictions.

We characterized the properties of editing substrates (Fig. 3c–f). The proximal and distal intronic ECSs are similar in length and max bulge size (Fig. 3d,e). Most of the ECSs are proximal and within 100 bp of the editing site (Fig. 3c), although there could be alternate, farther ECSs that we have not searched for. Most of the ECSs have 25–40 bp stems in which the largest bulge is 1–3 bp long (Fig. 3d,e). Notably, it seems that the editing site tends to be unpaired in comparison with ∼10 adjacent bases, which perhaps can aid ADAR's catalysis by making it easier to flip out an unpaired adenosine (Fig. 3f).

### Characterizing effects of edQTLs on edited dsRNA structures

We used the ECS predictions to characterize how edQTLs affect structures of RNA duplexes containing editing sites. Of the 545 total edQTLs identified, we were able to predict ECS locations for 276 of their associated editing sites. Of these 276 edQTLs, 45 lie within the edited dsRNA structure, either in the ECS or in the sequence surrounding the editing site that base pairs with the ECS. We also identified a set of 100 control variants that are not associated with editing level changes within the edited dsRNA structures (Methods section).

**Figure 4:**
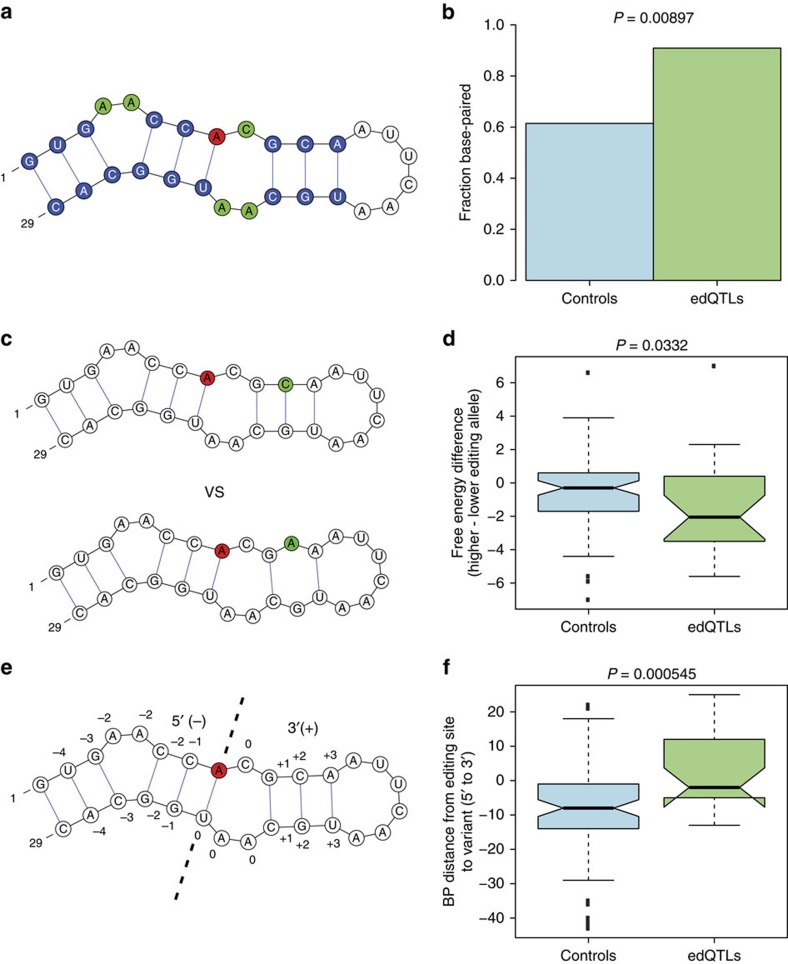
Effects of edQTLs on edited dsRNA structures. We looked at structural features that distinguish 27 edQTLs with effect size ⩾0.025 and 100 control variants in edited dsRNAs. (**a**) Cartoon dsRNA containing an editing site (red), base-paired nucleotides (blue) and non-base-paired nucleotides (green). (**b**) Fraction of edQTLs and control variants positioned at base-paired nucleotides. edQTLs are significantly more likely than control variants to be base-paired (Fisher's exact test). (**c**) Cartoon depicting the comparison of dsRNA free energies between the two alleles for a hypothetical variant. (**d**) Difference in dsRNA free energies between the two alleles for edQTLs and control variants, calculated as the free energy of lower edited allele subtracted from the higher edited allele. The edQTLs have a significantly larger difference in free energy than the control variants, with the higher editing allele having lower (more stable) free energies (one-sided Mann–Whitney *U*-test). (**e**) Same cartoon dsRNA as in (**a**) showing base pair distances between stem nucleotides and the editing site. The editing site is centred at position zero and the portion of the editing site side of the stem transcriptionally downstream of the editing site as well as its paired portion of the ECS was classified as positive. (**f**) Base-pair distances from edQTLs and control variants to the editing site. The control variants tend to be significantly enriched at the 5′ end of the dsRNA (one-sided Mann–Whitney *U*-test).

We looked for structural features that differ between the edQTLs and control variants within the edited dsRNAs. We restricted our analysis to 27 out of the 45 edQTLs that had an effect size of 0.025 or greater (5% or greater difference in editing levels between the two homozygotes), because we did not expect to see major differences induced in RNA structure by edQTLs with very low effect sizes. We hypothesized that dsRNA stability may influence the editing efficiency of ADAR to its target substrates. To test this hypothesis, we looked at two RNA features, effect on base pairing and duplex free energy. We noticed that QTL variants were more likely than control variants to be affecting nucleotides that are base paired (Fig. 4a,b). Base pairing of nucleotides within the dsRNA are important determinants of its stability and disruption of base pairing will change dsRNA stability. To expand upon this finding, we calculated the free energy of the two different alleles for each variant (Methods section). A more stable dsRNA structure will have a lower free energy and a presumably higher ADAR-binding affinity. We find that QTL variants are more likely than control variants to have a noticeable free energy difference between the two alleles, and for QTL variants, the allele with higher editing levels generally has a lower free energy, indicative of increased stability (Fig. 4c,d). We also looked at the location of variants within the edited dsRNA. We separated the RNA duplex into two regions, the portion of the duplex transcriptionally upstream of the editing site (5′) and the portion transcriptionally downstream (3′). We find that edQTLs tend to be very close to the editing site with a location distribution centred at the editing site as well as skewed towards the 3′ side of the duplex (Fig. 4e,f). On the other hand, control variants tend to be located at the 5′ side of the duplex. These same structural trends are also seen when we use the entire set of 45 edQTLs within edited dsRNAs (Supplementary Fig. 11).

### Identification of secondary *cis*-elements

**Figure 5:**
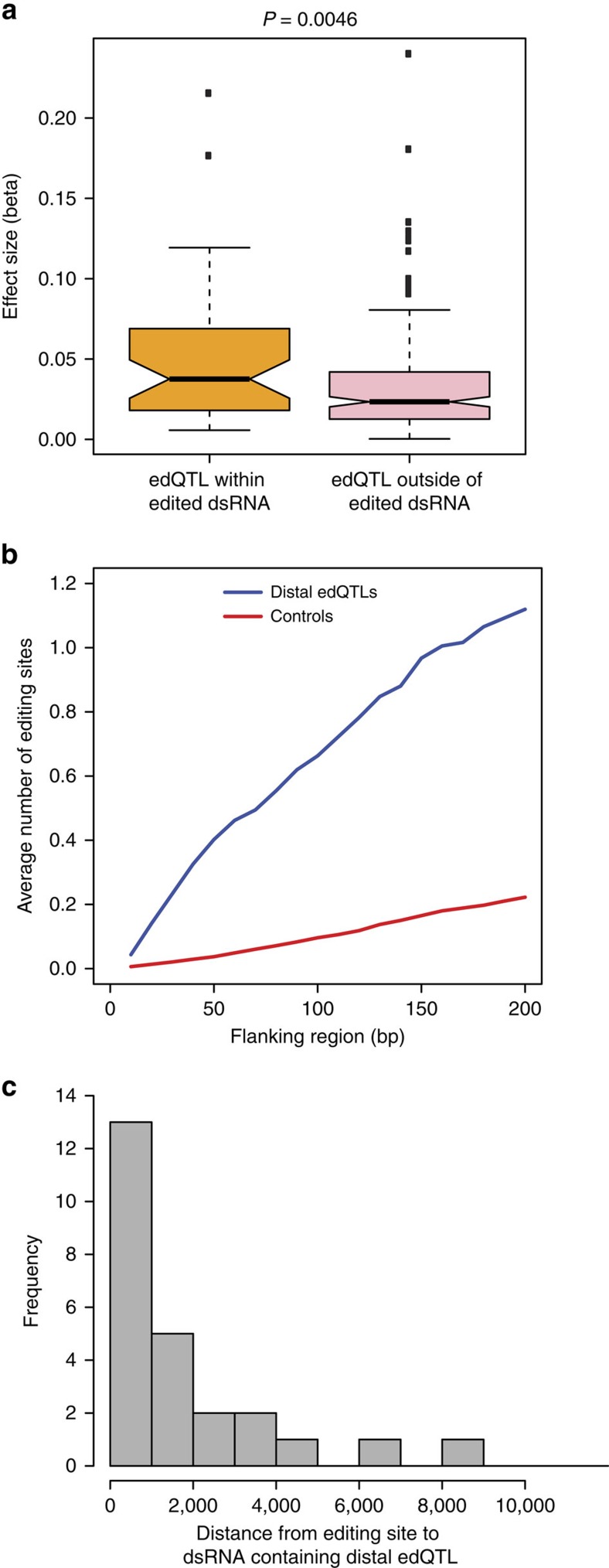
Characterization of distal edQTLs. (**a**) QTL effect sizes for 45 proximal edQTLs within the edited dsRNA and 213 distal edQTLs outside of the edited dsRNA. Effect sizes were calculated as half of the absolute value of the difference in average editing levels between the two homozygotes for each edQTL. Proximal edQTLs have significantly greater effect sizes (one-sided Mann–Whitney *U*-test). (**b**) Frequency of RNA editing sites nearby 213 distal edQTLs and 4247 matched control variants. For each variant, we calculated the number of RNA editing sites within sequence windows centred on the variant. (**c**) Distances from the editing sites to 28 dsRNA stems around distal edQTLs.

The majority of edQTLs, 213 (77%), lie outside of the edited dsRNA substrate. Not surprisingly, these ‘distal' edQTLs have smaller effect sizes than edQTLs within the edited dsRNA (Fig. 5a). However, these distal edQTLs are still located close to their associated editing site or close to other ADAR targets in the same gene (Fig. 5b). Recently, it has been discovered that additional nearby dsRNA stems modulate editing efficiencies of edited dsRNA substrates, mainly through enhancing recruitment of ADAR proteins^15^,^16^. One possible mechanism by which these distal edQTLs may be affecting editing is through changing RNA structure of one of these nearby dsRNA stems. We sought to identify these modulating dsRNA stems by predicting RNA structure around the distal edQTLs. We folded the sequence of the region 200 bps up- and downstream of distal edQTLs and matched control variants, similar to our Approach 1 for ECS predictions (Methods section). We identified 28 dsRNA stems with at least 20 base pairs and a maximum bulge size of 8 bp (Supplementary Fig. 12 and Supplementary Table 2). We found enrichment of dsRNA stems for distal edQTLs within 2 kb of the editing site (Fig. 5c), supporting the notion that additional dsRNA stems nearby the editing duplex can influence editing efficiency.

## Discussion

The *cis*-regulatory architecture of RNA editing is largely unexplored. The mechanisms targeting ADAR proteins to specific adenosines in an imperfect dsRNA are not well characterized, especially *in vivo*. In this study, we quantified RNA editing in natural strains of *D. melanogaster* using mmPCR-seq and used these editing level measurements to identify genetic variants associated with the differences in editing levels between strains. These edQTLs allowed us to identify structural features within the edited dsRNA duplex important for ADAR efficiency. In addition, distal edQTLs located outside of the primary dsRNA duplex guided us to locate secondary dsRNA stems that modulate editing.

We utilized mmPCR-seq to overcome the inherent biases of RNA-seq towards highly expressed genes. Using mmPCR-seq, we can efficiently capture and sequence up to 605 different loci from 48 different samples on a single microfluidic chip. We ran all PCR reactions to saturation, which provides uniform capturing efficiencies for the majority of targeted loci^21^ and we were able to achieve high accuracy in our editing level measurements (Supplementary Fig. 1). Using these accurate editing level measurements, we were able to identify 545 edQTLs.

RNA secondary structure is an important determinant of RNA editing specificity^14^ and we characterized the effect of edQTLs on RNA structure. To achieve this goal, we first had to systematically predict the locations of ECSs. In comparison with previous case studies^23^,^24^,^25^,^26^, our comprehensive analysis became possible through the development of an analytical framework to examine dsRNA structures. We showed that dsRNA stability is important for ADAR editing efficiency by demonstrating that variants reducing dsRNA stability tend to diminish editing (Fig. 4d). We also showed that variants in the dsRNA region 3′ of the editing site tend to affect editing levels, suggesting that the proximal 3′ region is important for ADAR binding (Fig. 4f).

Previous reports have implicated secondary dsRNA elements that influence editing at a nearby dsRNA^15^,^16^. The current hypothesized mechanism is that these dsRNA stems recruit ADAR proteins into the vicinity of the transcript. Using distal edQTLs located outside of the primary edited dsRNA, we were able to identify 28 of these secondary dsRNA stems. However, there are 185 distal edQTLs that do not lie within secondary dsRNA stems, suggesting unknown regulatory mechanisms in addition to RNA structure that remain unidentified.

We anticipate the application of edQTL mapping to human RNA editing. Human genome sequencing has identified many disease-associated variants, but their functional interpretation is challenging. Dysregulation of RNA editing has been implicated in a myriad of human diseases such as amyotrophic lateral sclerosis (ALS)^28^, autism^29^ and cancer^30^. The application of this methodology to human RNA editing will facilitate assignment of functional roles to disease-associated variants that affect RNA editing.

## Methods

### Collection of *D. melanogaster* strains

Fly stocks were reared at 25 °C. RNA was extracted from whole bodies of 3–5-day-old adult males for 131 DGRP^22^ strains in biological replicates. We excluded strains that were removed from DGRP v2 (ref. 31) as well as strains that had high identity by descent^32^.

### mmPCR-seq data generation and analysis

We quantified RNA editing at 605 loci using a multiplex microfluidic PCR with deep sequencing method developed in our lab^21^. We analysed two biological replicates for each of the 131 strains. In brief, we designed 48 pools of 12–13 plex multiplex PCR primers to amplify 605 loci. The sizes of the amplicons range from 150 to 350 bp. We loaded cDNAs and primer pools into the 48.48 Access Array IFC (Fluidigm) and performed target amplification as previously described^21^. PCR products of each sample were then subjected to a 15 cycle barcode PCR and pooled together. All pools were combined at equal volumes and purified via QIAquick PCR purification kit (Qiagen). The library was sequenced using Illumina HiSeq with 101 bp paired-end reads.

Paired-end reads were combined and mapped onto the genome (dm3) using BWA samse allowing 9 mismatches per read^33^. We aligned the sequencing reads to a combination of the reference genome and 100 bp exonic sequences surrounding known splicing junctions from available gene models (obtained from the UCSC genome browser). We quantified editing levels of known *D. melanogaster* RNA editing sites^3^ by taking the fraction of reads containing a ‘G' nucleotide at that position. For editing level quantification, sites covered by ⩾50 mmPCR-seq reads were used. For each strain, we excluded editing sites where the measured editing levels in the two biological replicates differed by >20% (see Supplementary Fig. 1c,d). Custom scripts used to process data are available upon request.

### RNA editing QTL mapping

For QTL mapping, we examined 789 RNA editing sites with editing level measurements in at least 35 strains. For each of the 789 RNA editing sites we normalized the editing level measurements. First, we centred and scaled each measurement by subtracting out the mean editing level value and dividing by the s.d. Then we quantile normalized the distribution to fit a standard normal distribution.

The following protocol was performed to map edQTLs genome-wide: (1) For each editing site, we fit linear models without any covariates between normalized editing levels and genotypes of each variant in the genome using Plink^34^. We only used variants in which the minor allele is present in at least four of the strains with an editing level measurement. We identified genome-wide edQTLs using a significance threshold of 1e−8 (Bonferroni method).

The following protocol was performed to map edQTLs in cis^35^: (1) For each editing site, we fit linear models without any covariates between normalized editing levels and genotypes of each variant in the same gene as that editing site using Plink^34^. We only used variants in which the minor allele is present in at least four of the strains with an editing level measurement. (2) We record the minimum *P* value (*P*_min_) across all variants tested for that particular editing site. (3) We repeat steps (1) and (2) for 10,000 permutations of the genotype sample labels and obtain 10,000 null *P* values (*P*null_1_, *P*null_2_,.. *P*null_10,000_). (4) We estimate an empirical *P* value for the most significant variant by determining where p_min_ lies within the null distribution (*P*null_1_–*P*null_10,000_). QTLs were called at FDRs of 10 and 5%, which were determined using the qvalue software^36^. For editing sites with a primary edQTL, to identify additional variants associated with RNA editing (secondary QTLs), we regressed out the effect of the primary edQTL and reran the linear models and permutations as described above. The effect sizes for each edQTL were calculated as one half of the difference in mean editing level between the two homozygotes using the original, non-normalized editing level values.

### Identification of intronic RNA editing sites

We obtained *D. melanogaster* yellow white (yw) strain nascent RNA-seq and ADAR null mutant nascent RNA-seq from NCBI SRA (GSE37232)^8^. We adopted a pipeline that can accurately map RNA-seq reads to the genome^27^. In brief, we used BWA^33^ to align RNA-seq data to a combination of the reference genome and exonic sequences surrounding known splicing junctions from available gene models (obtained from the University of California, Santa Cruz (UCSC) genome browser). We chose the length of the splicing junction regions to be slightly shorter than the RNA-seq reads to prevent redundant hits. After mapping, we used SAMtools^37^ to extract uniquely mapped reads, merged uniquely mapped reads of individual data sets from the same sample, and detected nucleotide variants between the RNA-seq data and reference genome. We took variant positions from the yw strain in which the mismatch was supported by ⩾2 reads and both base and mapping quality scores were at least 20. We required a minimum variant frequency of 3%. We used additional filters to remove wrongly assigned mismatches as previously described^27^. In brief, we removed mismatches in the first six bases of each read, simple repeats, homopolymer runs and those near splicing junctions. We also ensured that reads containing mismatches were uniquely mapped using BLAT^38^. We inferred the strand information of the sites based on the strand of the genes. Regions with bidirectional transcription (sense and antisense gene pairs) were discarded. ANNOVAR was used to annotate the editing sites^39^. Intronic sites that did not have altered reads in the nascent RNA-seq from ADAR null mutant flies were considered to be genuine A-to-I RNA editing sites.

We validated three randomly chosen intronic RNA editing sites by Sanger sequencing of RNA and DNA from heads of adult yw *D. melanogaster* flies. We used the same primers to amplify both cDNA and DNA: chr2L_2784071 5′- GAGGAATTTGCTTGCTGTGG -3′ and 5′- TACCCAAATGCCAACACAGA -3′, chr3L_4431719 5′- AGGATAACCCGGTCACACAC -3′ and 5′- GAACCGCTCGATTGTGGTAT -3′, chr3L_11546420 5′- TATTGACGACGACCTGCAAC -3′ and 5′- CCACTTTGCCGTGTTCTCTT -3′. For both RNA and DNA samples, we performed PCR using the KAPA SYBR Fast qPCR Kit (Kapa Biosystems) and the following protocol: initial enzyme activation at 95 °C for 3 min and 40 cycles of 95 °C for 3 s and 60 °C for 50 s. For RNA samples, we performed ribosomal RNA depletion^40^ and treated with Turbo DNase (Life Technologies) before reverse transcription (iScript Advanced, Bio-Rad). As a negative control, we also performed PCR for RNA samples without reverse transcription.

### Prediction of ECSs

To predict proximal ECSs, we predicted the secondary structure of the region within 200 bp of each editing site using the programs partition, MaxExpect, and ct2dot from the RNAStructure package^41^. The predicted ECS-like sequence is the sequence complementary to the editing site flanking sequence in the stem. We defined the stem as a region containing the editing site in which there was a stretch of base pairs with a defined max bulge size. The beginning and end of the ECS are the first and last bases that are paired in the stem, respectively. We then filtered our predicted ECSs to only include stems with lengths of at least 20 bp and a max bulge size of 8 bp, because these parameters yielded the greatest number of ECSs with high accuracy as estimated by the editing site enrichment in predicted ECSs (Supplementary Fig. 10).

To predict distal, intronic ECSs in *D. melanogaster*, we first identified conserved intronic regions as candidates. We smoothed phastCons scores using a sliding window of 51 bp (ref. 26). We selected regions that were within 2,500 bp of the editing site and at least 20 bases long with a smoothed phastCons score of at least 0.90 (determined using known intronic ECSs). Next, we obtained the candidate sequences for secondary structure predictions; we included a 30 base buffer on each side of these candidate regions and joined this to the region within 60 base of the editing site using a 100 base linker of adenosines. Then, we folded these sequences and identified ECSs as described above with the proximal ECSs, except that we searched for base pairing between the editing site and the candidate regions instead of flanking regions.

### RNA structure analysis of edQTLs

To compare against edQTLs in edited dsRNAs, we identified a set of control variants in edited dsRNAs that do not affect editing levels. This set of 100 control variants consists of all variants that were not edQTLs and were not in linkage with an edQTL (*R*^2^≤0.05). For each single nucleotide variant (edQTLs and controls) we used the Fold and ct2dot programs from RNAstructure^41^ to fold the two different alleles. Each allele consisted of the sequences for the editing side of the stem and the ECS joined together with a 100 bp linker of adenosines. For the analyses looking at fraction of variants base paired and location of variants in relation to the editing site (Fig. 4b,f), we identified the location and base-pairing status of the variant nucleotide using the structure of the allele with higher editing.

### Identification of secondary *cis*-elements

To predict dsRNA stems around distal QTLs and matched controls, as with the ECS predictions, we predicted the secondary structure of the region within 200 bp of each variant using the programs partition, MaxExpect, and ct2dot from the RNAStructure package^41^. We identified stems with lengths of at least 20 bp and a max bulge size of 8 bp similar to the ECS predictions. The matched control variants consisted of 4,247 randomly chosen variants within the same genes as the distal edQTLs that were not in the primary edited dsRNA duplex and were not in linkage with an edQTL (*R*^2^≤0.05).

## Additional information

**Accession codes:** The mmPCR-seq data was deposited to Gene Expression Omnibus at the National Center for Biotechnical Information under the accession number GSE67082.

**How to cite this article:** Ramaswami, G. *et al*. Genetic mapping uncovers *cis*-regulatory landscape of RNA editing. *Nat. Commun.* 6:8194 doi: 10.1038/ncomms9194 (2015).

## Supplementary Material

Supplementary Information Supplementary Figures 1-12, Supplementary Tables 1-2 and Supplementary References

Supplementary Data 1 mmPCR-seq primer sequences

Supplementary Data 2 RNA editing QTLs

Supplementary Data 3 novel intronic RNA editing sites

Supplementary Data 4 editing complementary sequence predictions
