# Supplementary material for: Genetic mapping uncovers cis-regulatory landscape of RNA editing
Source: Nat Commun. 2015 Sep 16;6:8194. doi: 10.1038/ncomms9194 (PMC4573499; doi:10.1038/ncomms9194)
Supplement: Supplementary Information — Supplementary Figures 1-12, Supplementary Tables 1-2 and Supplementary References [file ncomms9194-s1.pdf]

Supplementary Figures

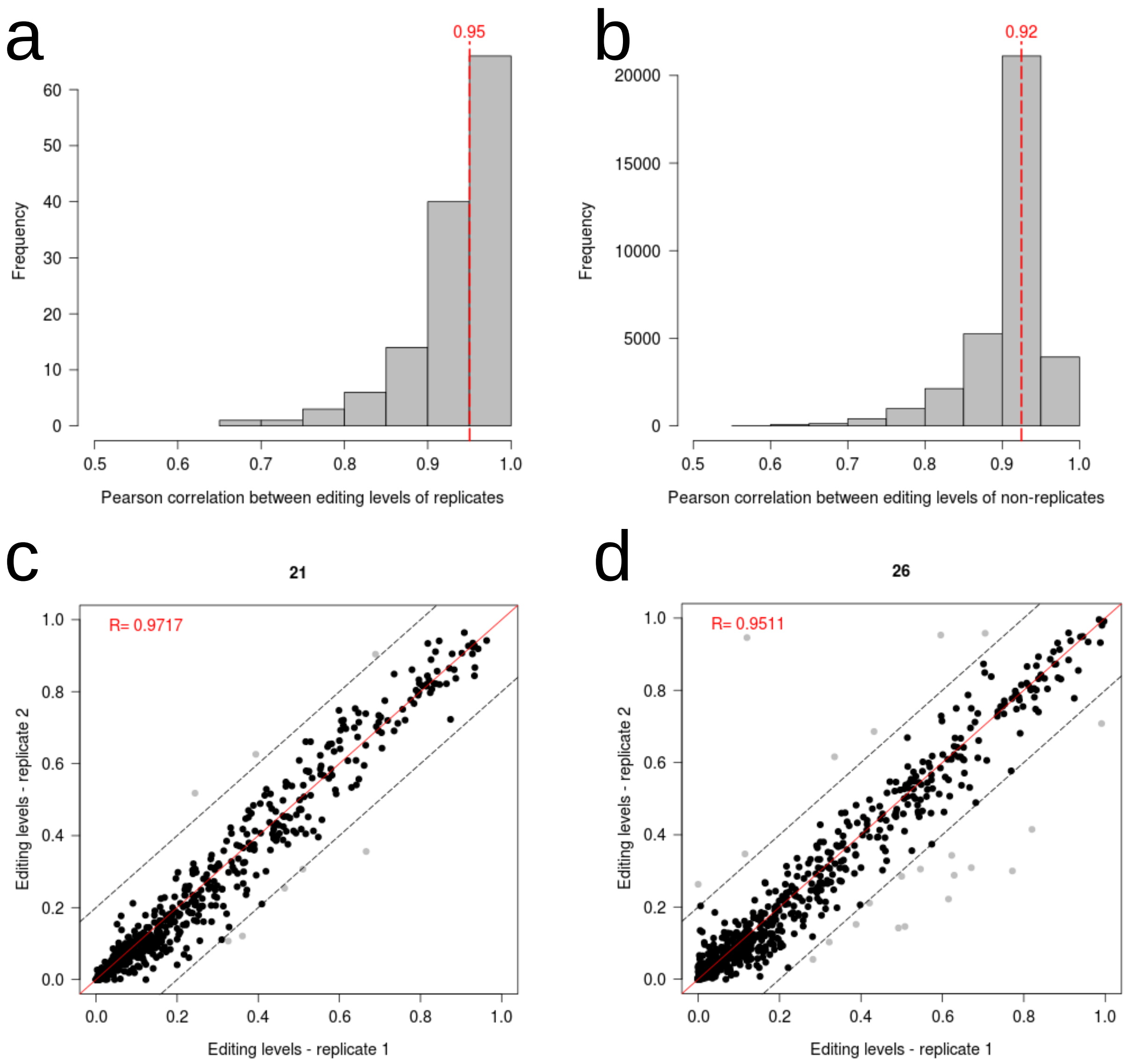

Supplementary Figure 1 - mmPCR-seq data for biological replicates. Pairwise spearman correlations between all **(a)** biological replicate and **(b)** non-replicate samples. The dashed red lines represent median values. Representative scatterplots of the editing levels measured in replicate 1 and replicate 2 for strains **(c)** 21 and **(d)** 26. Points shaded in grey had greater than 20% editing level difference between biological replicates and were excluded from further analysis.

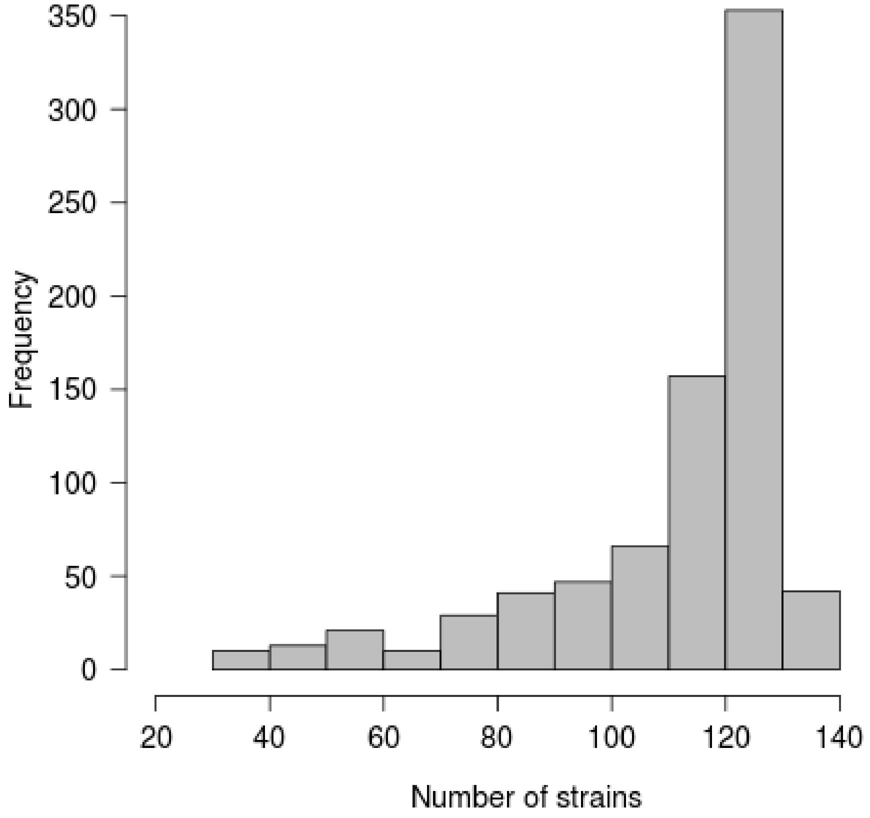

Supplementary Figure 2 – Number of DGRP strains with measurements at 789 editing sites. Histogram of number of DGRP strains with editing level measurements for each of the 789 editing sites.

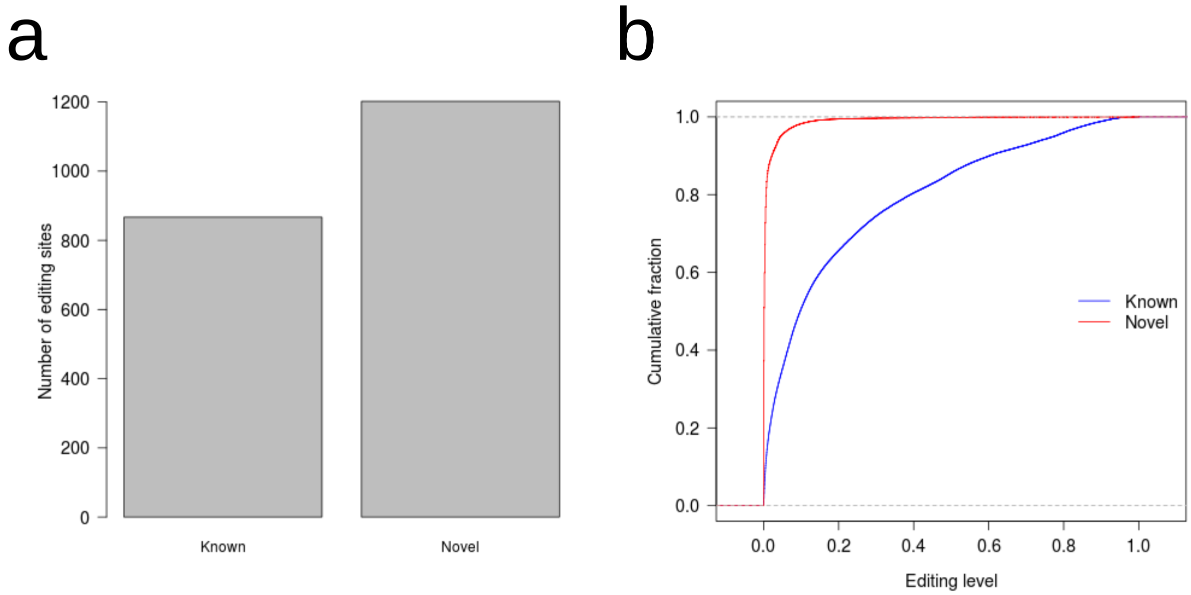

Supplementary Figure 3 - Identification of novel RNA editing sites. We used mmPCR-seq to identify novel RNA editing sites in the targeted loci. For each sample, we set a variant frequency cutoff to ensure at least 90% of the called variants were A-to-G or T-to-C mismatches. **(a)** The number of known and novel RNA editing sites in the targeted loci. **(b)** Cumulative distribution of editing levels for the known and novel editing sites.

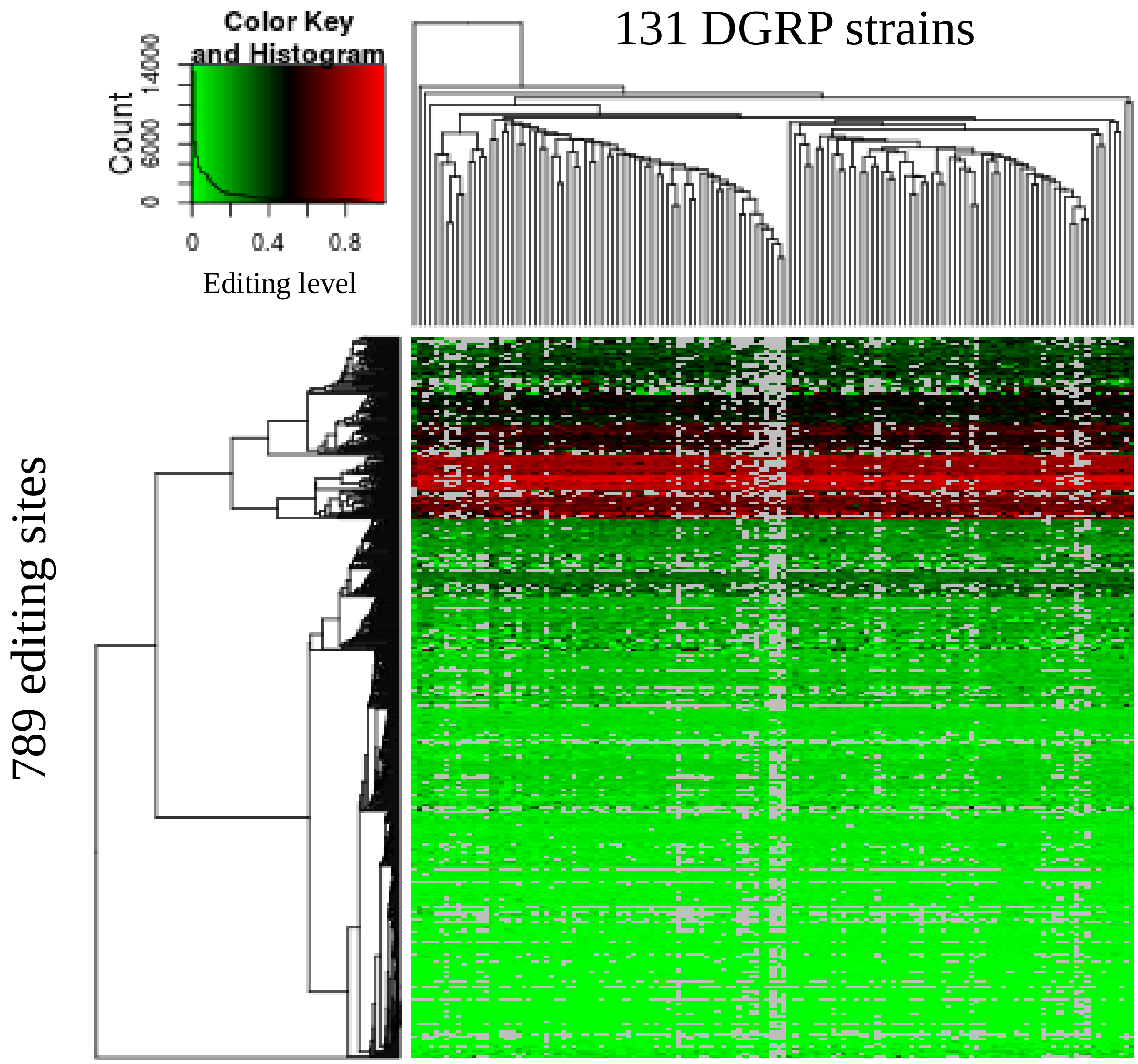

Supplementary Figure 4 – Quantification of editing levels at 789 sites in 131 DGRP strains. Heatmap of editing levels at 789 sites in 131 strains measured by mmPCR-seq. Editing sites without measurements are colored in grey.

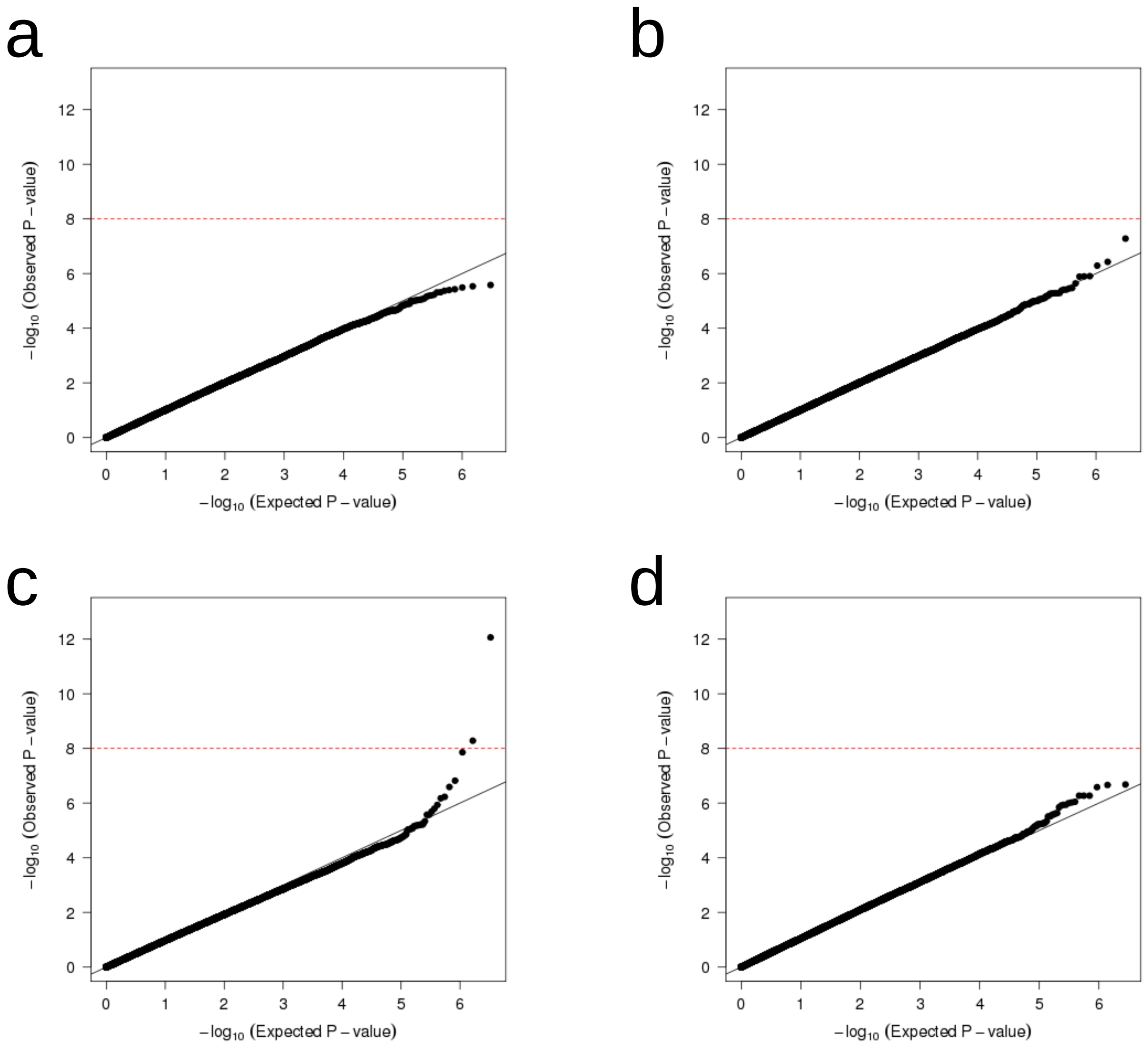

Supplementary Figure 5 – Genome wide edQTL mapping. QQ plots showing the distribution of test statistics for associations between editing levels and genotypes for all variants genome-wide for (**a**) chr2L:12724493 (**b**) chr2L:4986891 (**c**) chr2L:3503650 and (**d**) chr2L:283707. Red dashed lines represent a genome wide significance threshold of 1e-8 (Bonferroni correction).

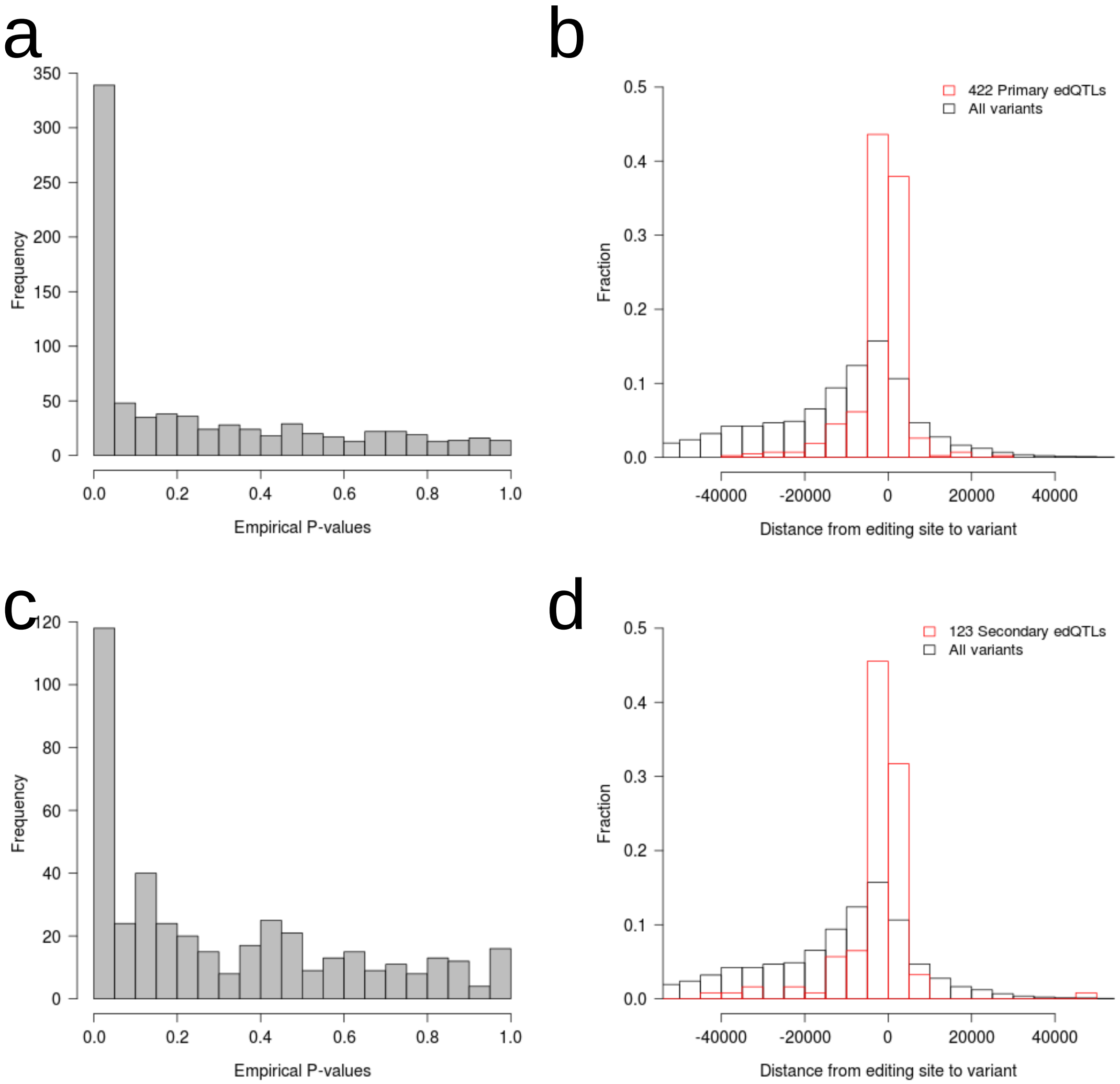

Supplementary Figure 6 - RNA editing QTL mapping. (a,c) Empirical P-value distributions for the most strongly associated variant for each RNA editing site based on 10,000 random permutations. **(a)** Primary edQTL mapping for 789 RNA editing sites and **(c)** secondary edQTL mapping after regressing out the effect of the primary edQTL for 422 editing sites. **(b,d)** Distance from the editing site to the edQTL is plotted for **(b)** primary edQTLs and **(d)** secondary edQTLs.

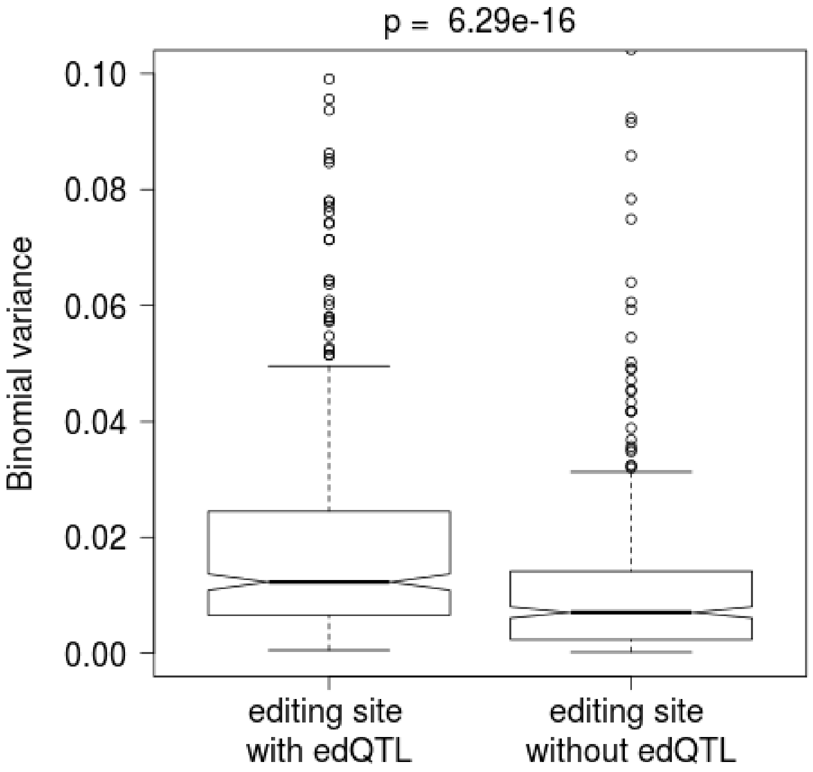

Supplementary Figure 7 – Variability in editing levels. Binomial variance values for editing sites with an edQTL vs editing sites without an edQTL (one sided Mann-Whitney U-test).

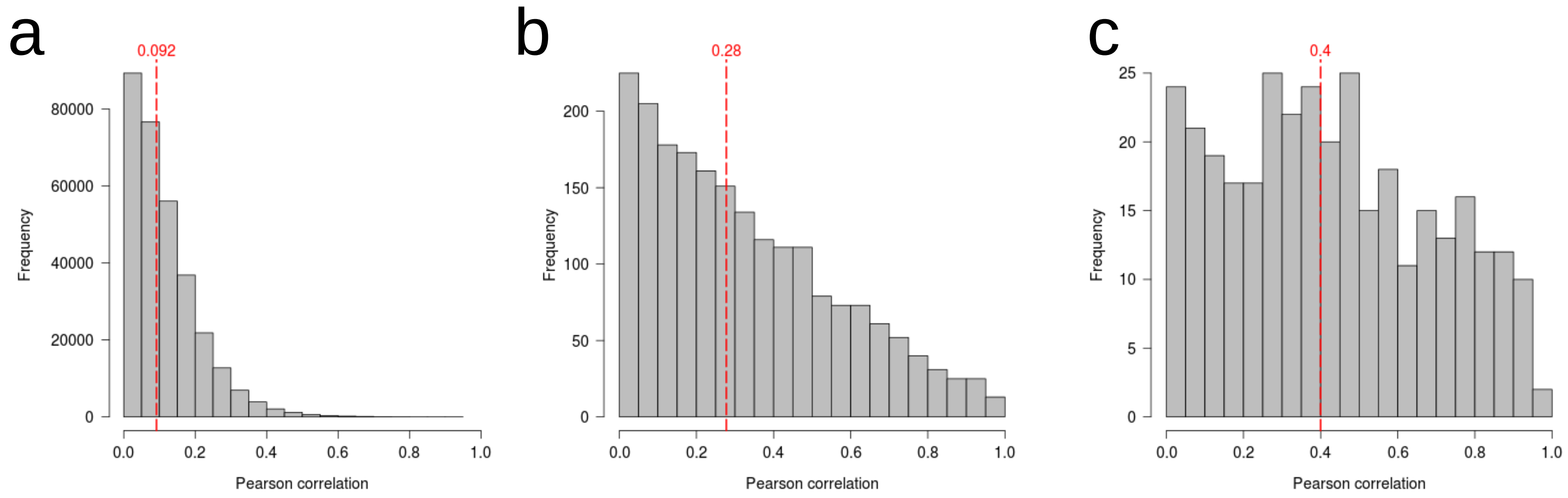

Supplementary Figure 8 - Pairwise correlations of RNA editing sites. Pairwise spearman correlations between editing sites in **(a)** different genes, **(b)** in the same gene, and **(c)** in the same dsRNA duplex. Dashed red lines correspond to the median values.

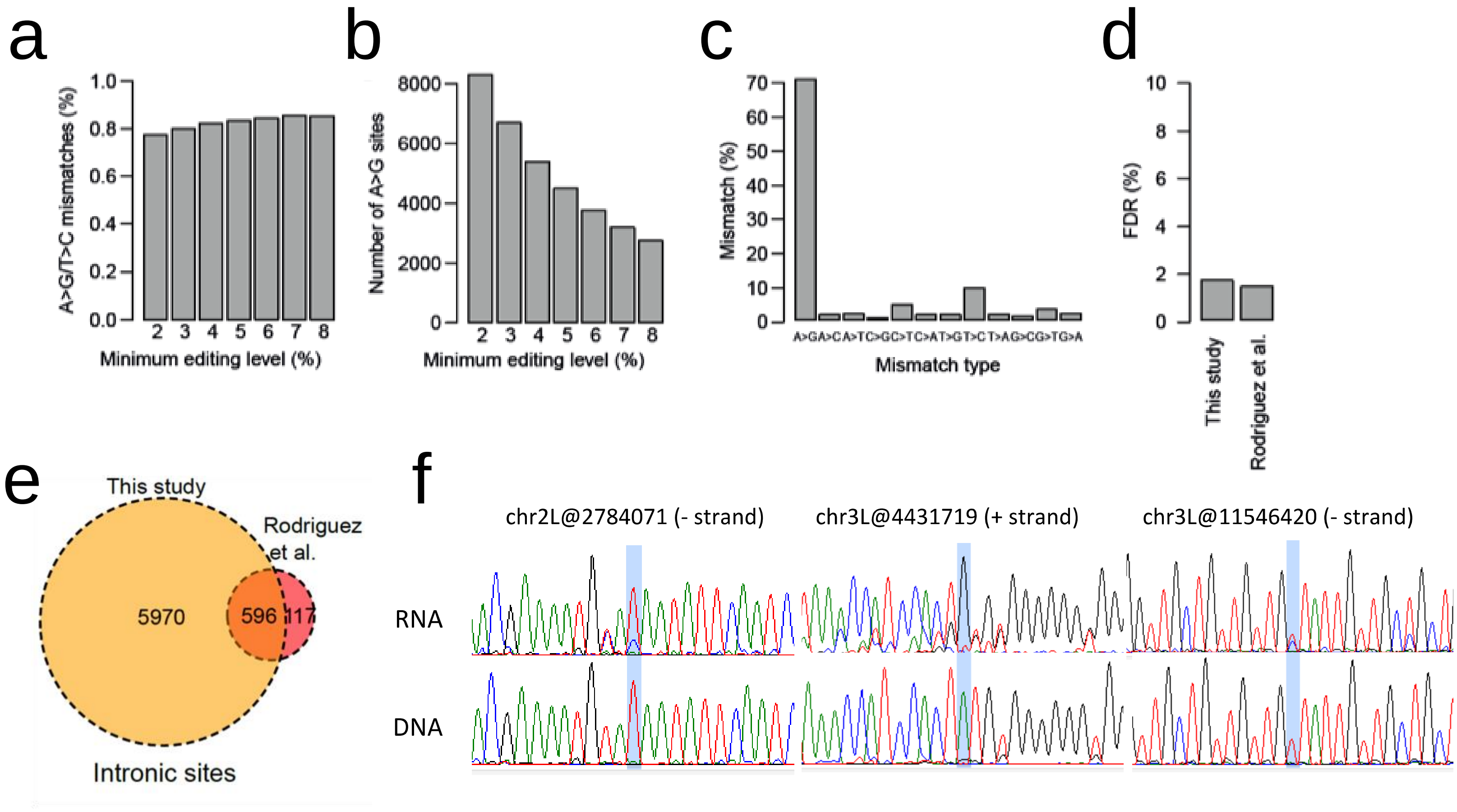

Supplementary Figure 9 - Accurate identification of intronic RNA editing sites in *D.melanogaster*. (a) Relationship between the proportion of detected mismatches that are A-to-G/T-to-C and the minimum editing level cutoff (i.e., the percentage of reads with the variant nucleotide). **(b)** Relationship between the number of A-to-G variants and the minimum editing level cutoff. **(c)** Percentage of RNA variants of all 12 mismatch types. **(d)** The FDR of intronic RNA editing site identification for the 2 specified studies. FDR was calculated by identifying A-to-G mismatches present in nascent RNA-seq from ADAR null flies. **(e)** Overlap between intronic RNA editing sites found in this study and those found previously. (**f**) Sanger sequencing validation of three randomly chosen novel intronic editing sites (highlighted in blue).

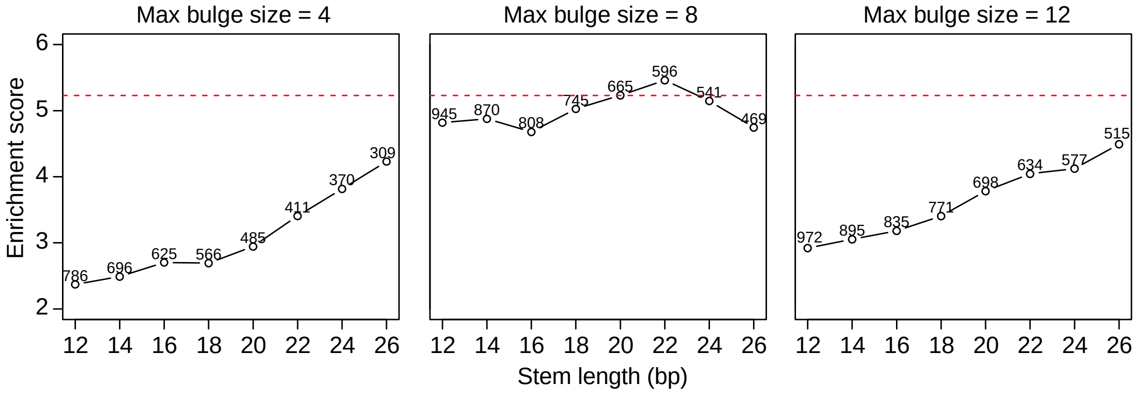

Supplementary Figure 10 - Selecting cutoffs for editing complementary sequence (ECS) predictions. The enrichment of editing sites in predicted ECS regions relative to the nearby control regions (**Fig. 3a**), with various min stem length and max bulge size cutoffs. The enrichment score is the number of editing sites in predicted ECSs divided by that in control regions. The number of ECS regions predicted for each cutoff is indicated on the plot. The red dotted line indicates the enrichment score of the cutoff finally selected for use.

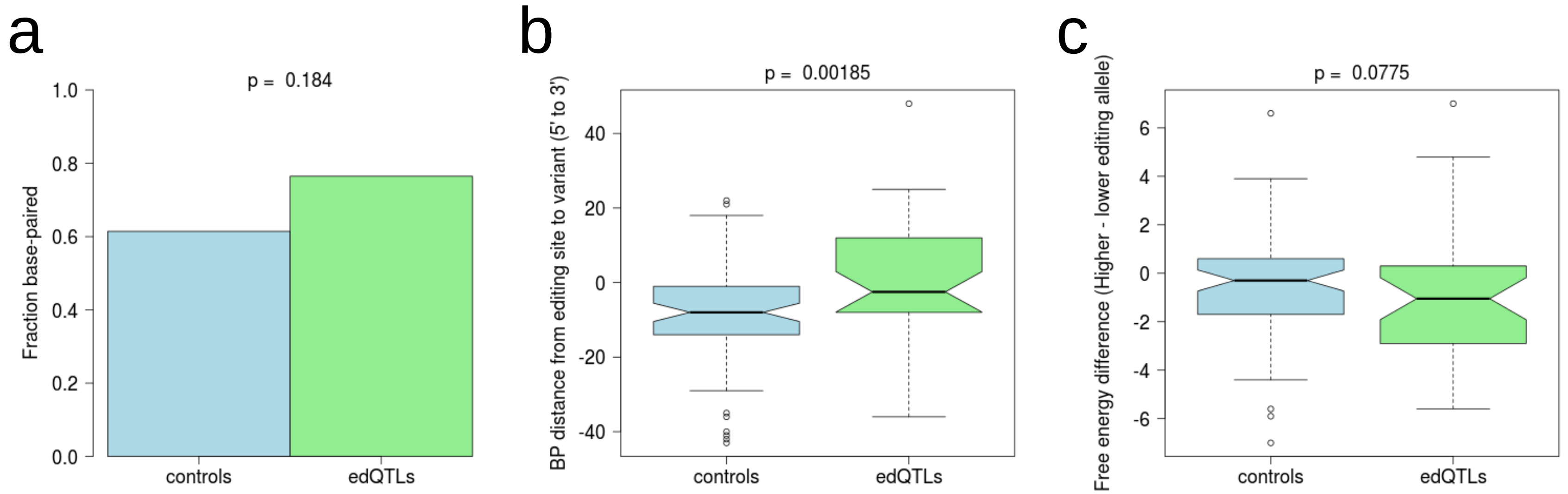

Supplementary Figure 11 - Effects of edQTLs on edited dsRNA structures. Same plots as Figure 4b,d,f except using all edQTLs. **(a)** Fraction of edQTLs and control variants positioned at base-paired nucleotides (Fisher's exact test). **(b)** Base-pair distances from edQTLs and control variants to the editing site (one-sided Mann-Whitney U-test). **(c)** Difference in dsRNA free energies between the two alleles for edQTLs and control variants, calculated as the free energy of lower edited allele subtracted from the higher edited allele (one-sided Mann-Whitney U-test).

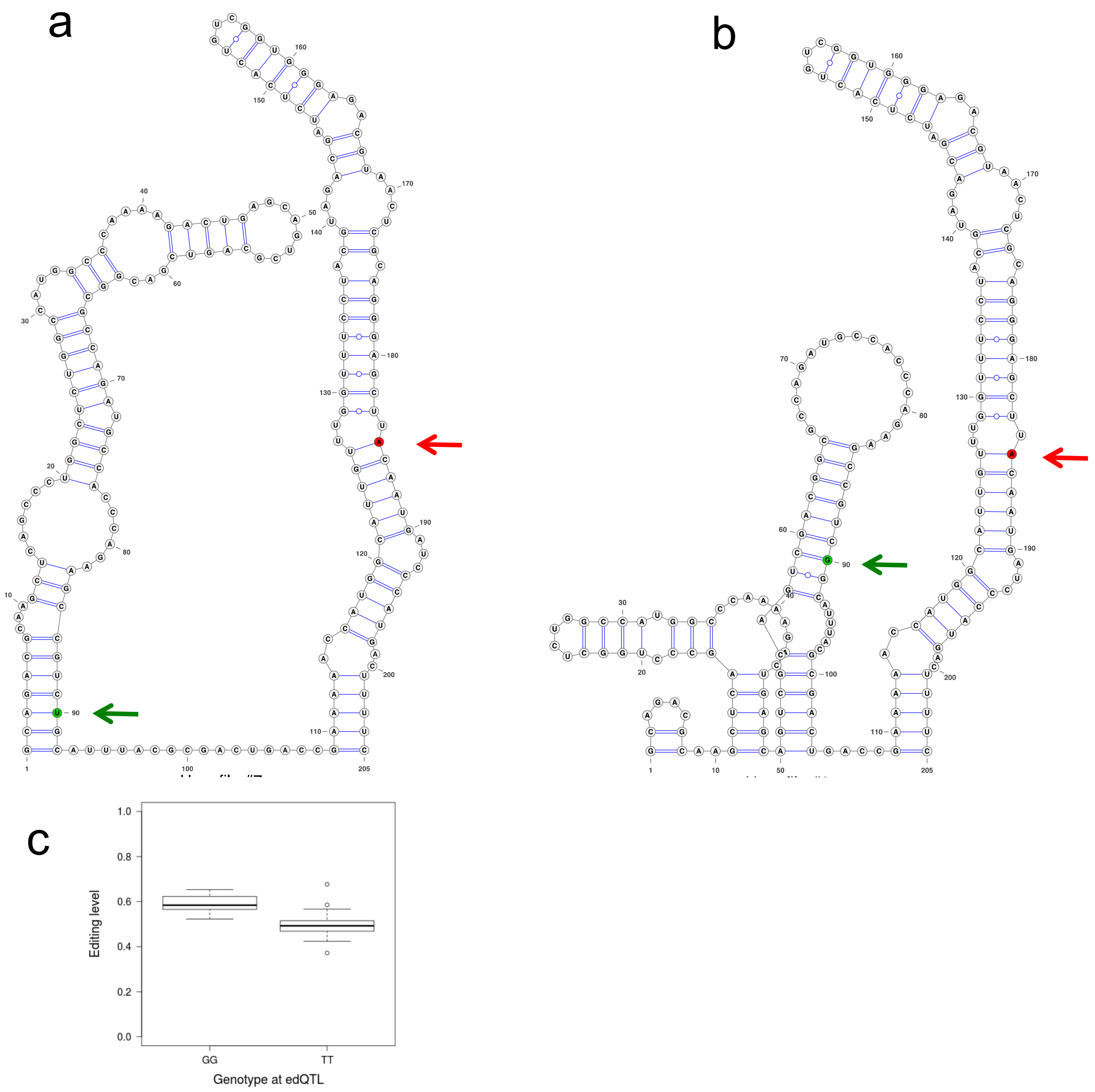

Supplementary Figure 12 – Distal edQTL in secondary dsRNA stem modulates editing in the NaCP60E gene. (a,b) RNA secondary structure predictions for the **(a)** T allele and the **(b)** G allele. The red arrow points towards the editing site (highlighted in red) and the green arrow points towards the distal edQTL (highlighted in green). **(c)** Relationship between editing levels and strain genotypes for the edQTL in NaCP60E.

Supplementary Tables

Supplementary Table 1 - Summary of previously identified ECSs in *Drosophila melanogaster*.

Chrom	Gene	Gene name	Exonic edited positions	Edited stem start	Edited stem end	ECS start	ECS end	Note	Reference
chr2L	CG3139	Synaptotagmin 1	2785542	2785510	2785546	2784048	2784086	pseudoknot	[1]
chr2L	CG3139	Synaptotagmin 1	2785605, 2785606, 2785610, 2785621	2785595	2785625	2784345	2784376	pseudoknot; part of the above structure	[1]
chrX	CG9907	Paralytic	16367661, 16367670, 16367671	16367658	16367675	16366514	16366536		[2]
chrX	CG9907	Paralytic	16376715, 16376716, 16376726	16376700	16376735	16375881	16375919		[3]
chrX	CG12348	Shaker	17824684, 17824687, 17824691	17824667	17824694	17826897	17826927		[4]
chrX	CG12348	Shaker	17824766	17824757	17824785	17825734	17825760		[4]
chrX	CG12348	Shaker	17844072, 17844090	17844062	17844092	17842741	17842772		[4]

Supplementary Table 2 – dsRNA stems identified around distal edQTLs

Chrom	Position	Strand	complement_start	complement_end	QTLduplex_start	QTLduplex_end
chr3R	23498276	+	23498339	23498364	23498253	23498279
chr3L	9826538	+	9826574	9826603	9826537	9826564
chr3R	27668532	+	27668333	27668358	27668516	27668539
chr2R	20798251	+	20798162	20798207	20798216	20798253
chr2L	8202442	-	8202395	8202428	8202438	8202469
chr3R	23499447	+	23499541	23499566	23499436	23499464
chr3R	21792114	+	21792065	21792105	21792113	21792150
chr2L	16176451	+	16176473	16176496	16176441	16176465
chr3R	20492805	+	20492837	20492881	20492789	20492828
chr3L	9826547	+	9826574	9826603	9826537	9826564
chr2L	8202440	-	8202394	8202428	8202438	8202470
chr2R	9698348	+	9698472	9698502	9698326	9698355
chrX	1272323	+	1272500	1272523	1272308	1272329
chr2R	4757819	+	4757844	4757882	4757802	4757839
chr2R	20798580	+	20798387	20798411	20798571	20798602
chrX	14723771	-	14723708	14723740	14723748	14723780
chr2R	9067191	-	9067029	9067066	9067189	9067219
chrX	19536050	-	19536091	19536133	19536047	19536086
chr3R	4666009	-	4665915	4665948	4665975	4666010
chr3R	20533219	+	20533385	20533416	20533199	20533229
chr2R	18277541	+	18277469	18277515	18277528	18277575
chr3L	21323854	+	21323893	21323931	21323853	21323888
chrX	17880884	-	17880767	17880820	17880858	17880913
chr2R	20307749	-	20307687	20307728	20307733	20307782
chrX	6007239	-	6007115	6007149	6007221	6007251
chr3R	23499562	+	23499436	23499464	23499541	23499566
chr2L	6453262	-	6453340	6453419	6453262	6453334
chr3R	19900751	-	19900838	19900899	19900747	19900804

Supplementary References

1. Reenan RA. Molecular determinants and guided evolution of species-specific RNA editing. *Nature* 434, 409-413 (2005).

2. Hanrahan CJ, Palladino MJ, Ganetzky B, Reenan RA. RNA editing of the Drosophila para Na(+) channel transcript. Evolutionary conservation and developmental regulation. *Genetics* 155, 1149-1160 (2000).

3. Reenan RA, Hanrahan CJ, Ganetzky B. The mle(napts) RNA helicase mutation in drosophila results in a splicing catastrophe of the para Na+ channel transcript in a region of RNA editing. *Neuron* 25, 139-149 (2000).

4. Ingleby L, Maloney R, Jepson J, Horn R, Reenan R. Regulated RNA editing and functional epistasis in Shaker potassium channels. *The Journal of general physiology* 133, 17-27 (2009).
